# Efficacy of Hand Gestures and the Hand as Foot Teaching Method Compared to Traditional Methods in Medical and Dental Education: A Scoping Review

**DOI:** 10.1002/jdd.70190

**Published:** 2026-02-25

**Authors:** Shivangi Vats, Manuel S. Thomas, Vinod Jathanna

**Affiliations:** ^1^ Department of Conservative Dentistry and Endodontics Manipal College of Dental Sciences Mangalore Manipal Academy of Higher Education Manipal India

**Keywords:** dental education, didactic methods, embodied learning, gesture‐based learning, hand gestures, hand‐as‐Foot teaching method, medical education, scoping review

## Abstract

**Objective:**

The “Hand‐as‐Foot” teaching method is an innovative, hands‐on approach that uses gesture‐based movements to represent anatomical structures and functions. It has been used successfully in medical education to help simplify and communicate complex ideas. However, its use in dental education remains relatively unexplored. This scoping review set out to examine how hand gestures, including the Hand‐as‐Foot technique, are being used in medical and dental classrooms, and whether they offer benefits over traditional teaching methods. We also aim to identify gaps in the current research to guide future studies.

**Methods:**

We followed the PRISMA‐ScR guidelines to conduct a comprehensive literature search in MEDLINE (PubMed) and SCOPUS, with additional manual searches in Google Scholar and key academic journals. Studies were included if they evaluated the effectiveness of hand gestures in teaching clinical or non‐clinical subjects in medical or dental education. Eligible study designs included clinical trials, interventional studies, observational studies, and cohort or case‐control studies. Only English‐language publications were included, while reviews, editorials, and opinion pieces were excluded. A descriptive analysis was used to summarize the findings.

**Results:**

Out of 57 records identified, 29 were duplicates, 15 were removed, and 14 were resolved manually leaving 42 articles for screening. After title and abstract review, 10 articles were excluded. Full‐text screening of 32 articles led to the removal of 26 that were out of scope. In total, six studies were included in the review. Of these, four were comparative studies that directly evaluated gesture‐based teaching against traditional lecture‐style instruction in medical and dental education.

**Conclusion:**

Although early findings are promising, research on gesture‐based teaching especially the “Hand‐as‐Foot method” is still limited, particularly in dental education. Most available studies suggest improved engagement and understanding, but high‐quality, comparative research is needed to support broader adoption of these techniques in health education.

## 1 Introduction

Traditional didactic teaching methods, though foundational, increasingly demonstrate limitations in actively engaging students and fostering the comprehensive skill set essential for contemporary clinical practice in medicine and dentistry [1]. The effectiveness of the hand gesture analogy teaching method is supported by a robust scientific basis that integrates three primary theoretical foundations. This comprehensive approach draws on principles from cognitive science, educational psychology, and nonverbal communication (NVC) [1, 2]. The cognitive foundation is rooted in contemporary neuroscience and educational psychology research. It states that the processing of gestures activates multiple neural networks simultaneously, engaging both the motor and cognitive areas of the brain [3]. Multimodal activation is believed to enhance the encoding and retrieval of information by creating robust neural pathways [3]. Hand gestures also function as physical anchors for abstract concepts, thereby creating concrete representations that help bridge the gap between theoretical understanding and practical application in medical education [3]. Meta‐analyses show that medical students who used gesture‐based instruction improved their spatial reasoning skills and their retention of procedural knowledge when compared to students using traditional learning methods [3].

Designing effective visual mnemonics in medicine and dentistry is crucial for enhancing the learning of complex morphological and anatomical concepts [4]. There are multiple forms of mnemonics, including auditory, visual, and kinesthetic types. Visual mnemonics can simplify intricate structures, making them more accessible and memorable for students. This approach is particularly beneficial in fields like medicine and dentistry, where understanding spatial relationships and anatomical details is essential. Hand mnemonics represent a simple visual technique that can facilitate clearer and more effective learning [5]. Since medical subjects involve a large volume of information, the use of mnemonic methods can be particularly advantageous. For example, cardiac cycle hand mnemonic could help students grasp the concept more easily, as it links key information with visual and physical cues, thereby supporting memory retention [6, 7]. This strategy can be readily implemented in classroom teaching, making conventional lectures on the cardiac cycle more interactive and efficient [8]. The integration of hand gestural teaching techniques in educational settings has gained attention for its potential to enhance learning experiences, in complex subjects like medical and dental education [9, 10, 11]. This approach leverages the natural human ability to use gestures for communication and understanding, making abstract concepts more tangible and accessible. There are various innovative teaching methods, including the “Hand as Foot” teaching method and the “Pen and Palm” model, which use everyday gestures and objects to facilitate learning [3].

There is evidence supporting the potential benefits of using hand gestures to enhance the learning of anatomy, particularly for medical students. One study introduced the concept of “Air Anatomy,” where simple hand gestures were combined with lectures on complex spatial anatomical concepts, such as the anatomy of extraocular muscles. This randomized controlled trial revealed that students who learned with the inclusion of hand gestures scored significantly higher in post‐tests for both recall and application of knowledge compared to those who received traditional lectures only. Importantly, students with low to moderate spatial ability benefited the most from the use of hand gestures, reporting lower cognitive load and an improved overall classroom experience [10]. This suggests that hand gestures can be a cost‐effective and accessible method to aid spatial understanding, a common challenge in anatomy education.

Broadly, hand gestures have been recognized as an effective communication tool across different sectors, including education and medicine. A proposed system to recognize and use hand gestures for conveying complex information showed high accuracy in supporting communication among teachers, students, and medical professionals, indicating its utility in learning environments [12]. This generalized utility further supports the concept that hand gestures could have positive effects beyond anatomy learning in medical fields. Even though there is a clear appreciation among medical students for interactive and contextual learning methods such as body painting, which allows active, embodied learning experiences, evidence on direct comparison between hand gestures and other modalities in anatomy or other fields is limited [13]. Furthermore, studies on anatomy learning strategies have noted that successful education involves a balance between memorization, understanding, and visualization. Hand gestures likely support the visualization and understanding components by offering a dynamic and spatially grounded way to engage with anatomical content [14]. While this is well documented in medical education, exploration of hand gestures use in anatomical or spatial learning in non‐medical fields remains less established but may follow similar principles.

### 1.1 Hand‐as‐Foot Technique in Dentistry

In this technique, the hand is shaped and manipulated to mimic the oral cavity, including lip movements, teeth positions, and sometimes tongue or jaw motion. This tactile and visual representation helps learners better understand the spatial relationships and dynamic movements within the mouth, facilitating more concrete comprehension compared to purely verbal or static visual explanations. The hand‐as‐foot technique simulates oral structures or functions using the educator's hand as a visual and kinesthetic model of the mouth. Although predominantly employed in clinical teaching in medical and dental education to illustrate oral anatomy and function, the “hand‐as‐foot” technique also shows potential beyond clinical contexts. It can be used for patient education, helping patients understand procedures or pathological conditions more clearly. However, this technique remains less explored and formalized within dental education despite the clear utility in clinical skill development and communication. In dental education specifically, where understanding intricate oral structures and functions is crucial, the “hand as foot” or “hand as mouth” approach offers a cost‐effective, low‐tech complementary method to traditional teaching tools such as extracted teeth, wax carving, and digital models. As dental anatomy education progresses to integrate digital and hands‐on active learning strategies, the “hand as foot” method can enrich the psychomotor and cognitive learning experiences by providing immediate, personalized spatial, and functional demonstrations [15, 16].

#### 1.1.1 Objective

The “Hand as Foot Teaching Method” has been effectively employed in medical education to simplify and convey key and complex concepts. This innovative approach, which utilizes hand gestures to simulate anatomical structures and functions, holds potential for application in dental education as well. The objective of this scoping review is to explore the extent to which this method has been utilized in dentistry and to evaluate its potential role in enhancing learning outcomes in dental classrooms. In addition, this review seeks to identify existing gaps in the scientific literature regarding the application of the Hand‐as‐Foot technique, thereby laying the groundwork for its broader integration across various disciplines within dental education by answering following questions (Table 1):

1. What is the extent of use of hand‐gesture‐based instructional methods, including structured approaches such as the Hand‐as‐Foot technique, in medical and dental teaching settings worldwide?
2. What evidence exists on the educational value of hand‐gesture‐based teaching methods, such as the Hand‐as‐Foot Technique, in supporting comprehension and retention among medical and dental learners?

**TABLE 1:** Review question, PCC approach, search strategy in the different databases evaluated, and the inclusion/exclusion criteria.

Review question	1. What is the extent of use of hand‐gesture‐based instructional methods, including structured approaches such as the Hand‐as‐Foot Technique, in medical and dental teaching settings worldwide?
	2. What evidence exists on the educational value of hand‐gesture‐based teaching methods, such as the Hand‐as‐Foot Technique, in supporting comprehension and retention among medical and dental learners?
PCC approach	*Population‐ P*: Dental and medical undergraduate and post graduate students.
	*Concept‐ C*: Hand gestures, hand as foot teaching method used in medical and dental education by educators.
	*Context‐C*: Comparison of structured hand gestures against traditional didactic teaching methods.

**Database**	**Search strategy**
PubMed	(“dental student”[tiab] OR “medical student*”[tiab]) AND (gesture*[tiab] OR “hand gesture*”[tiab] OR finger*[all] OR “hand as foot*”[tiab] OR “hand as foot*”[tiab]) AND (traditional*[tiab] OR didactic*[all] OR lecture*[tiab]) (“*Dental student*”[tiab] OR “medical student*”[tiab]) AND (Gesture*[tiab] OR “Hand Gesture*”[tiab] OR finger* OR “Hand as foot*”[tiab] OR Hand‐as‐foot*[tiab]) AND (Traditional*[tiab] OR didactic* OR lecture*[tiab])
Scopus	(TITLE‐ABS(“dental student”) OR TITLE‐ABS(“medical student*”)) AND (TITLE‐ABS(gesture*) OR TITLE‐ABS(“hand gesture*”) OR ALL(finger*) OR TITLE‐ABS(“hand as foot*”) OR TITLE‐ABS(“hand as foot*”)) AND (TITLE‐ABS(traditional*) OR ALL(didactic*) OR TITLE‐ABS(lecture*)) (TITLE‐ABS(“*Dental student*”) OR TITLE‐ABS(“medical student*”)) AND (TITLE‐ABS(Gesture*) OR TITLE‐ABS(“hand gesture*”) OR finger*OR TITLE‐ABS(“hand as foot*”) OR TITLE‐ABS(Hand‐as‐foot*)) AND (TITLE‐ABS(Traditional*) OR didactic* OR TITLE‐ABS(lecture*))
Eligibility criteria	Clinical trials, interventional studies, case‐control studies, cohort studies, empirical, and observational studies conducted in dental and medical education settings, evaluating the efficacy of hand gestures in teaching clinical and non‐clinical disciplines of medicine and dentistry, were included. Literature reviews, letters to the editor, and opinion pieces were excluded.

## 2 Methods

Clinical trials, interventional studies, case‐control studies, cohort studies, and observational studies conducted in dental and medical education settings, evaluating the efficacy of hand gestures in teaching clinical and non‐clinical disciplines of medicine and dentistry, were included in this scoping review. Only studies published in English were considered. Literature reviews, letters to the editor, and opinion pieces were excluded. This review adhered to the PRISMA‐ScR (Preferred Reporting Items for Systematic Reviews and Meta‐Analyses extension for Scoping Reviews) guidelines [17]. The primary sources of information included bibliographic databases: *MEDLINE (PubMed)*, and *SCOPUS (Elsevier)*. Additional studies were identified through manual searches in *Google Scholar* and relevant high‐impact journals. The search strategy was developed using a combination of keywords, MeSH terms, and synonyms (Table 1). A descriptive analysis was performed to summarize and interpret the findings.

### 2.1 Study Selection Process

All records identified through the database searches were first imported into Mendeley Reference Manager to remove duplicates. The deduplicated list of articles was then exported to the Rayyan platform for screening. Two reviewers independently screened the titles and abstracts of the articles using the predefined eligibility criteria. Full‐text screening of included and uncertain records was also conducted independently by the same two reviewers, following the same inclusion and exclusion criteria. Any discrepancies at either stage, title/abstract or full text, were resolved through discussion and consensus with a third reviewer.

### 2.2 Data Charting Process

Two reviewers collaboratively developed a data‐charting form using Microsoft Excel (Microsoft Corporation, Redmond, WA, USA) to capture key variables relevant to the scoping review questions. The form was iteratively refined through discussion and consensus during the initial phase of data extraction. Both reviewers then independently charted the data, with ongoing comparison and refinement to ensure consistency. A third reviewer performed a double‐check of the extracted data for accuracy. Any discrepancies were addressed through discussion between the primary reviewers, and if consensus could not be reached, a third reviewer provided adjudication.

## 3 Results

### 3.1 Selection and Characteristics of Sources of Evidence

**FIGURE 1:**
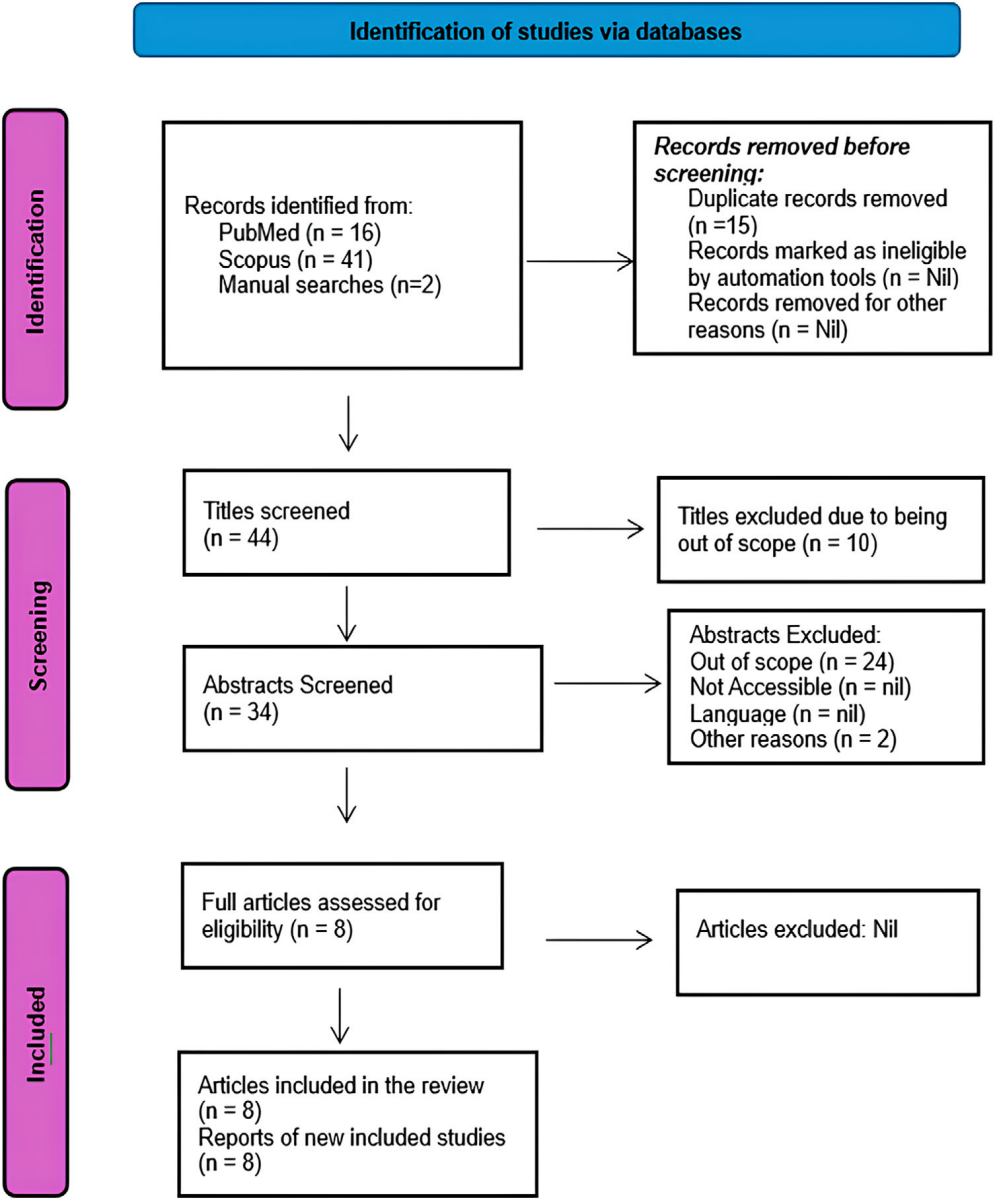
Prisma flow diagram.

A total of 57 records were identified through database and manual searches. Among these, 29 duplicate records were detected; of these, 15 were removed, while 14 were resolved through manual verification. Following de‐duplication, 42 records were screened by title and abstract, leading to the exclusion of 10 articles. Upon full‐text review, 24 articles were excluded for being out of scope. Two studies that fell within the scope of this review were found to be published in two separate journals under different titles, despite presenting identical data and findings. In accordance with ethical standards and to maintain methodological integrity as outlined in the PRISMA guidelines, both instances of this duplicate publication were excluded from the final analysis. Ultimately, eight articles met the inclusion criteria and were included in the final scoping review (Figure 1 ). Among the included clinical and pedagogically oriented studies, four were comparative evaluation studies and randomized trials that assessed the effectiveness of gestural teaching techniques in comparison to traditional didactic learning in medical and dental education (Table 2). The remaining were observational and empirical studies.

**FIGURE 2:**
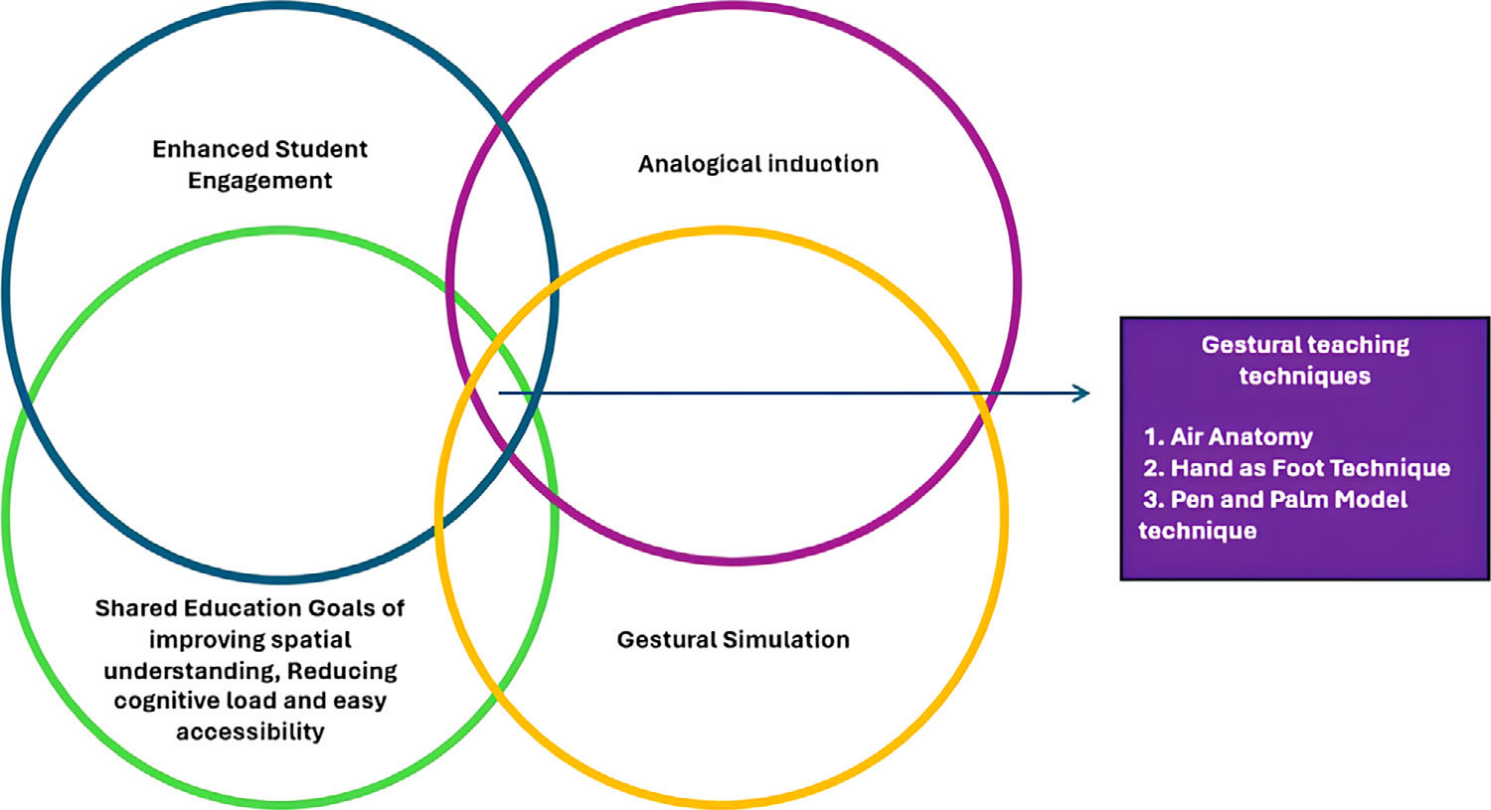
Similarities of various gestural teaching methods in medical education.

**TABLE 2:** Information on the clinical studies included in this scoping review.

S. no.	References	Objectives	Type of study	Type of intervention	Main findings
1.	He et al. [3]	The applicability of gesture‐based learning in clinics for learning motion system injury	Descriptive study	Hand as foot teaching method	The method was welcomed by medical students and achieved good teacher–student interaction
2.	Xuan et al. [9]	To understand the learning outcomes in neuroanatomy after using Graven database in addition to traditional teaching techniques	Observational study	Graven database includes pictures of hand gestures representing anatomy, nerves, and vessels.	Significant improvement in the academic performance of students using the Graven database combined with traditional techniques
3.	Yohannan et al. [10]	To explore whether a technique called "Air Anatomy," which uses deictic and iconic hand gestures, can enhance spatial anatomical understanding among medical students.	Randomized control trial	Air anatomy—hand gestures	The intervention group showed significantly higher post‐test scores for basic recall and applied knowledge. Students with low to moderate spatial ability benefited most. The technique reduced extrinsic cognitive load and increased germane cognitive load.
4.	Su et al. [11]	To assess the effectiveness of the “Hand as Foot” teaching method in comparison to traditional teaching methods within the “Human Physiology” course, specifically focusing on the “Urinary System”	Randomized Control Trial	Hand as Foot teaching method	The experimental group reported significantly higher ratings across all dimensions of teaching effectiveness, including engagingness, intuitiveness, facilitation of understanding, enhancement of memorization, and effortlessness of learning, compared to the traditional teaching group.
5.	Yang et al. [18]	To investigate the impact of the "hand as foot" teaching method on students' knowledge related to Parkinson's disease (PD)	Comparative/quasi‐experimental study	Hand as Foot teaching method	*Student Perception of Usefulness*: A significant majority (75.61%) of students in Class 1 (who used the “hand as foot” method) found the method helpful *Impact on Knowledge Correctness*: The “hand as foot” teaching method significantly improved the correctness of knowledge related to PD *Specific Knowledge Gains*: Students taught with the “hand as foot” method showed high correct answer rates for key PD concepts. *General Reception and Benefits*: The method was generally well‐received by medical students, fostering good teacher–student interaction and effective understanding and memorization of difficult knowledge points, receiving positive feedback from students.
6.	Ismail et al. [19]	Different non‐verbal communication (NVC) techniques in an online setting of medical lecture delivery.	Observational study	NVC (body position, facial expressions, voice intonation, body movements, eye contact, paralinguistics)	Consistent NVC patterns were identified, such as lecturers leaning forward, maintaining direct eye contact, and using dynamic voice intonation. Initial student apprehension turned positive upon observing cheerful facial expressions and intonation, fostering open reception of feedback and motivation to address communication skill deficiencies. Students also preferred comfortable learning environments.
7.	Varikasuvu et al. [20]	To propose teaching model, the “pen and palm analogy,” to help visualize and understand the complex theories of enzyme function, specifically the coexistence of the induced‐fit and substrate‐strain models of enzyme action	Conceptual model and pedagogical proposal	Pen and palm model: This method involves using common objects (a pen and a palm) to represent abstract biochemical concepts (enzyme, substrate, active site, induced‐fit, substrate‐strain, and product formation)	The core finding is that complex biochemical concepts, such as enzyme function, can be effectively visualized and made more accessible through the creative use of simple, everyday objects like a pen and a palm, aiming to enhance student engagement and comprehension. Further studies are suggested to determine the impact of the technique.
8.	Lisk et al. [21]	To evaluate the effectiveness of the anatomy glove learning system (AGLS)	Empirical study	AGLS, which consists of a glove imprinted with anatomically correct bones worn on the non‐dominant hand, and video clips demonstrating drawing structures onto the glove using colored markers	Knowledge of hand anatomy improved significantly in both groups immediately after learning. However, self‐confidence in the 3D group (AGLS) gradually increased over time, while in the 2D group, it began to decline 1 week later. Students perceived AGLS as a positive and helpful learning experience.

### 3.2 Summary of Results (Table 2)

These studies explored various NVC techniques and gesture‐based teaching strategies in the context of medical education, with most focusing on enhancing anatomical understanding, student engagement, and cognitive processing. Several studies demonstrated the positive impact of integrating hand gestures or gesture‐based analogies into teaching methods, For example, Graven database use combining hand gesture imagery with traditional teaching resulted in notable improvement in academic performance [9]. The “Hand as Foot” teaching method, featured in multiple studies consistently reported positive student perception, enhanced understanding, and memorization, and in controlled trials, statistically significant improvements in test scores and teaching effectiveness compared to traditional methods [3, 11, 18]. The “Air Anatomy” technique using deictic and iconic hand gestures, was found to be particularly effective for students with low to moderate spatial ability, reducing extraneous cognitive load and improving applied knowledge scores [10]. In one of the studies by Ismail et al., a broader set of non‐verbal cues including facial expressions, eye contact, body posture, and paralinguistic were evaluated in an online lecture setting. Results highlighted recognizable patterns of effective NVC such as dynamic voice tone and facial expressions, which contributed to a positive shift in student receptivity and motivation [19]. In addition, innovative physical analogies, such as the “pen and palm” model, were introduced to simplify complex biochemical theories, showing promise in conceptual visualization and student engagement, though further validation was recommended [20]. Finally, the Anatomy Glove Learning System (AGLS) proved beneficial in improving anatomical knowledge and self‐confidence. The 3D learning group using AGLS showed sustained confidence gains over time, in contrast to a decline in the traditional 2D learning group [21].

## 4 Discussion

The outcomes of this scoping review identified parallels that can be drawn in Gestural Teaching, including Air Anatomy, the Hand as Foot Method and NVC techniques by teachers. Both the “Air Anatomy” and “Hand as Foot” methods are innovative, gestural teaching techniques used in medical education to simplify complex anatomical and physiological concepts. They rely on using hands and body movements to create tangible, three‐dimensional representations of abstract or hard‐to‐visualize biological structures, moving beyond traditional two‐dimensional teaching aids like PowerPoint presentations [3, 9, 10]. The core principle of both methods is to make learning more intuitive, interactive, and memorable by turning abstract information into concrete, physical experiences [3]. Hand gestures in classes are mentioned as NVC skills encompassing body position, facial expressions, voice intonation, body movements, eye contact, and paralinguistic [19].

### 4.1 Core Similarities (Figure 2)

#### 4.1.1 Gestural Simulation

Both methods use hand gestures to simulate anatomical structures. “Hand as Foot” uses the upper limbs to demonstrate the anatomy of the lower limbs, leveraging the high structural similarity between them [3]. Similarly, “Air Anatomy” uses a combination of deictic (pointing) and iconic hand gestures to create an impression of anatomical structures “in the air.” For example, “Hand as Foot” might use a clenched fist to represent the femoral head, while “Air Anatomy” might use cupped hands to “grip” the thalamus [3, 11].

#### 4.1.2 Analogical Induction

A key concept in both approaches is analogical induction, where students are guided to understand complex systems by comparing them to more familiar ones. “Hand as Foot” explicitly encourages students to find “similarities and differences” between upper and lower limb structures and diseases [3]. “Air Anatomy” also relies on this principle, as students use their hands to create analogies for anatomical parts, which helps bridge the gap between abstract concepts and physical experiences [11].

#### 4.1.3 Enhanced Student Engagement

Both methods aim to transform passive learning into an active, participatory process. They enliven the classroom atmosphere, increase teacher‐student interaction, and encourage students to use their “hands and brains” [3, 11]. This active engagement helps capture students’ attention, foster interest, and improve comprehension and retention compared to traditional lecture‐based methods [3, 9]. Similarly, the “Pen and Palm” model uses a simple analogy involving a pen and a palm to represent enzymatic reactions, illustrating the coexistence of the induced‐fit and substrate‐strain theories of enzyme action. This model creatively integrates imaginative narratives into traditional teaching methods, helping students visualize enzyme function theories and enhancing their understanding and engagement with enzymatic reactions [20].

### 4.2 Shared Educational Goals

1. *Improving Spatial Understanding*: A primary goal for both is to help students develop a three‐dimensional mental picture of anatomical relationships, which is a common challenge in medical education. By physically representing structures, these methods aid in the spatial understanding of concepts that are often presented in flat, 2D formats [3].
2. *Reducing Cognitive Load*: By making abstract concepts more concrete and intuitive, both techniques can effectively reduce the extrinsic cognitive load—the mental effort required to process the way information is presented. This allows students to dedicate more cognitive resources to understanding the subject matter itself [3].
3. *Cost‐Effectiveness and Accessibility*: “Air Anatomy,” “Hand as Foot,” and “pen and palm model” are presented as accessible, “no‐cost” or low‐cost alternatives to expensive high‐tech teaching aids like virtual reality (VR) or 3D models. They can be implemented without special equipment, making them particularly valuable in educational settings with limited resources [3, 11].

In essence, “Air Anatomy,” “Hand as Foot” and NVC techniques are founded on the same pedagogical philosophy: using the body as a dynamic, interactive tool to make the invisible visible. While “Hand as Foot” focuses on a specific analogy (upper limb for lower limb), “Air Anatomy” is a broader application of gestural simulation for various anatomical concepts. Both, however, serve the ultimate purpose of making complex medical education more effective and engaging.

In this current review, most of the studies included were observational studies and randomized trials with mentioned interventions of teaching techniques and the data was collected using reflective writing. For specific analyses, students were tasked with writing reflections on their performance and lecturer feedback after small group discussions [9, 19]. The key findings are that regions with limited medical education resources, an image teaching method can address the scarcity and simplicity of traditional teaching aids while also reducing teaching expenses. However, other inferences can also be drawn, for example, visual mnemonics, shared learning goals as well as improved memory and recollection of the morphological and anatomical structures.

## 5 Creation of Effective Visual Mnemonics

### 5.1 Use of Physical Models and Gestures

Techniques like “Air Anatomy” utilize hand gestures to enhance spatial understanding of anatomical concepts. This method combines iconic gestures, eye gaze, and pointing to create a visual and interactive learning experience, which can be particularly beneficial for students with low to moderate spatial abilities [3, 10, 11, 18]. Low‐fidelity physical models, such as those used in the AGLS, allow students to engage in kinesthetic learning by drawing anatomical structures onto a glove. This hands‐on approach helps students understand the relationship between structure and function, making the learning process more interactive and memorable [21].

### 5.2 Simplification and Visualization

Methods like air anatomy and the Hand‐as‐Foot technique can be used to create visual mnemonics, bringing about a better understanding of morphological structures. Visual mnemonics aim to turn abstract and complex anatomical concepts into concrete and vivid representations. This can be achieved through the use of gesture‐based teaching methods that make learning more intuitive and engaging [3]. Effective visual mnemonics often involve breaking down complex structures into simpler components, using visual cues to highlight important details. This approach helps students focus on key aspects of the anatomy, facilitating better understanding and recall [19].

#### 5.2.1 Visual Mnemonics Integration With Technology

The development of databases, such as GRAVEN, which include hand gesture representations of neuroanatomy, offers an accessible and cost‐effective way to supplement traditional teaching methods. These resources can be used for both in‐class learning and self‐study, providing flexibility and reinforcing learning outside the classroom [9].

## 6 Broader Implications and Future Directions

The principles outlined that the combination of traditional teaching methods with innovative visual and interactive techniques can significantly enhance the learning of morphology and anatomy in medicine and dentistry. Future research could explore the integration of emerging technologies, such as augmented reality, to further improve the effectiveness of visual mnemonics. In addition, developing standardized guidelines for creating and implementing these tools could help educators maximize their potential benefits in diverse settings of dental education. While traditional multimedia methods have limitations, the integration of VR and 3D models can provide a more immersive learning experience. Technologies like interactive web 3D and Google Glass allow students to visualize anatomical structures in three dimensions, which can enhance their understanding and retention of complex information [9, 21, 22, 23].

Medical Teaching and communication can be enhanced by the use of the telestration technique where an instructor or clinician draws over a visual (e.g., radiographs, anatomy images, surgical video) in real‐time using a stylus, mouse, or touch screen [24]. This method can be employed in future dental education for better learning outcomes.

Another System, the Leap Motion Controller (LMC), developed by Ultraleap Ltd., is a low‐cost, contact‐free VR device designed to accurately capture hand and finger movements. Utilizing two cameras and three infrared sensors, the LMC connects via USB and does not require the placement of sensors on the user's body, making it a non‐invasive and user‐friendly option. It is capable of tracking the hand, wrist, and arm movements of up to four individuals simultaneously. Due to its compact size, ease of installation, and compatibility with a range of applications, the LMC has gained traction in both neurorehabilitation and educational contexts. In medical education, it facilitates user interaction within a three‐dimensional virtual environment and has been integrated into gamified learning modules and simulations. This makes it particularly valuable for subjects like neuroanatomy, where spatial understanding is critical. Studies have validated the LMC as a reliable and effective tool for enhancing engagement and comprehension in eLearning settings. In clinical practice, the device has also been employed in physical rehabilitation, supporting motor recovery exercises through immersive, gesture‐based interfaces [25].

### 6.1 Observational Limitations

Since, only English‐language studies and few databases were used, relevant literature published in other languages or platforms may have been missed. Apart from PubMed and Scopus, information in other electronic databases has not been included. Inconsistent terminology and methodology may affect the consistency and reproducibility of findings.

Lack of availability of comparative data since there is a scarcity of studies that directly compare the Hand as Foot method with conventional teaching techniques, making it difficult to draw conclusions about its relative effectiveness. For example, In the study by Ismail et al., the researcher's observations primarily recorded repetitive, long‐duration NVC, often described as unconscious habitual movements. The small sample size makes it difficult to draw conclusions that apply widely across different educational contexts or to compare NVC types between younger and more senior lecturers or across genders [19]. One of the clinical studies involving Graven database usage count, it was limited to undergraduate students, excluding neurosurgery residents, despite the database's potential utility for both groups [9]. They concluded gesture‐based teaching methods, such as the “Hand as Foot” method, to be interactive and participatory compared to traditional lecture‐based teaching, stimulating students' interest and making them more actively engaged in learning. Further investigation should also aim to determine the efficacy of the different components of the AGLS (glove vs. video) [21].

## 7 Conclusion

There is a notable lack of comparative studies evaluating the effectiveness of embodied learning methods, such as the Hand‐as‐Foot gesture‐based teaching approach, in contrast to conventional instructional techniques within medical and dental education. Most existing literature on hand gestures in education primarily emphasizes their role in enhancing conceptual understanding, memory retention, and learner engagement. The Hand‐as‐Foot method, in particular, remains a relatively novel and specialized strategy, frequently reported in the form of letters to the editor, pilot studies, or case‐based observations, most commonly in the context of anatomy education. High‐quality evidence, such as randomized controlled trials directly comparing this technique with traditional didactic methods, remains scarce, especially in dental education settings. Utilization of this technique has been restricted to medical education. There is scope for including Hand as foot technique in various disciplines of dental education as well.

## Funding

The authors have nothing to report.

## Disclosure

The authors have nothing to report.

## Ethics Statement

The authors have nothing to report.

## Consent

The authors have nothing to report.

## Conflicts of Interest

The authors declare no conflicts of interest.

## Supporting information




**Supporting Figure 1**: jdd70190‐sup‐0001‐PRISMA‐Checklist.docx
